# Association of Circulating Progesterone With Breast Cancer Risk Among Postmenopausal Women

**DOI:** 10.1001/jamanetworkopen.2020.3645

**Published:** 2020-04-24

**Authors:** Britton Trabert, Doug C. Bauer, Diana S. M. Buist, Jane A. Cauley, Roni T. Falk, Ashley M. Geczik, Gretchen L. Gierach, Manila Hada, Trisha F. Hue, James V. Lacey, Andrea Z. LaCroix, Jeffrey A. Tice, Xia Xu, Cher M. Dallal, Louise A. Brinton

**Affiliations:** 1 Division of Cancer Epidemiology and Genetics, National Cancer Institute, Bethesda, Maryland; 2 Department of Medicine and Department of Epidemiology and Biostatistics, University of California, San Francisco; 3 Kaiser Permanente Washington Health Research Institute, Seattle, Washington; 4 Graduate School of Public Health Department of Epidemiology, University of Pittsburgh, Pittsburgh, Pennsylvania; 5 Division of Cancer Etiology, Department of Population Sciences, Beckman Research Institute of City of Hope, Duarte, California; 6 Division of Epidemiology, Department of Family and Preventive Medicine, University of California, San Diego; 7 Leidos Biomedical Research Inc, Frederick, Maryland; 8 School of Public Health, University of Maryland College Park

## Abstract

**Importance:**

The role of endogenous progesterone in the development of breast cancer remains largely unexplored to date, primarily owing to assay sensitivity limitations and low progesterone concentrations in postmenopausal women. Recently identified progesterone metabolites may provide insights as experimental data suggest that 5α-dihydroprogesterone (5αP) concentrations reflect cancer-promoting properties and 3α-dihydroprogesterone (3αHP) concentrations reflect cancer-inhibiting properties.

**Objective:**

To evaluate the association between circulating progesterone and progesterone metabolite levels and breast cancer risk.

**Design, Setting, and Participants:**

Using a sensitive liquid chromatography–tandem mass spectrometry assay, prediagnostic serum levels of progesterone and progesterone metabolites were quantified in a case-cohort study nested within the Breast and Bone Follow-up to the Fracture Intervention Trial (n = 15 595). Participation was limited to women not receiving exogenous hormone therapy at the time of blood sampling (1992-1993). Incident breast cancer cases (n = 405) were diagnosed during 12 follow-up years and a subcohort of 495 postmenopausal women were randomly selected within 10-year age and clinical center strata. Progesterone assays were completed in July 2017; subsequent data analyses were conducted between July 15, 2017, and December 20, 2018.

**Exposures:**

Circulating concentrations of pregnenolone, progesterone, and their major metabolites.

**Main Outcomes and Measures:**

Development of breast cancer, with hazard ratios (HRs) and 95% CIs was estimated using Cox proportional hazards regression adjusted for key confounders, including estradiol. Evaluation of hormone ratios and effect modification were planned a priori.

**Results:**

The present study included 405 incident breast cancer cases and a subcohort of 495 postmenopausal women; the mean (SD) age at the time of the blood draw was 67.2 (6.2) years. Progesterone concentrations were a mean (SD) of 4.6 (1.7) ng/dL. Women with higher circulating progesterone levels were at an increased risk for breast cancer per SD increase in progesterone levels (HR, 1.16; 95% CI, 1.00-1.35; *P* = .048). The association with progesterone was linear in a 5-knot spline and stronger for invasive breast cancers (n = 267) (HR, 1.24; 95% CI, 1.07-1.43; *P* = .004). Among women in the lowest quintile (Q1) of circulating estradiol (<6.30 pg/mL) elevated progesterone concentrations were associated with reduced breast cancer risk per SD increase in progesterone levels (HR, 0.38; 95% CI, 0.15-0.95; *P* = .04) and increased risk among women in higher quintiles of estradiol (Q2-Q5; ≥6.30 pg/mL) (HR, 1.18; 95% CI, 1.04-1.35; *P* = .01; *P* = .04 for interaction).

**Conclusions and Relevance:**

In this case-cohort study of postmenopausal women, elevated circulating progesterone levels were associated with a 16% increase in the risk of breast cancer. Additional research should be undertaken to assess how postmenopausal breast cancer risk is associated with both endogenous progesterone and progesterone metabolites and their interactions with estradiol.

## Key Points

**Question:**

Are circulating levels of progesterone and progesterone metabolites associated with incident breast cancer in postmenopausal women?

**Findings:**

In this case-cohort study of 405 incident breast cancer cases, elevated circulating progesterone levels were associated with a 16% increase in the risk of breast cancer.

**Meaning:**

This study’s findings suggest that further research should be undertaken to assess how postmenopausal breast cancer risk is associated with both endogenous progesterone and progesterone metabolite levels, as well as their interactions with estradiol levels.

## Teaser

This case-cohort study examines the association of circulating progesterone and progesterone metabolite concentrations with breast cancer in postmenopausal women.

## Introduction

Although it is well accepted that pharmacologic and physiologic concentrations of estradiol are associated with increased mitogenic activity of breast epithelial cells, the possible association with progesterone and breast cancer risk continues to be debated.^1,2,3,4,5,6,7^ Studies reporting increased breast cancer risk with menopausal hormone therapy use have primarily evaluated exogenous synthetic progestogen (progestin) use,^8,9,10^ rather than endogenous progesterone. In addition to possible progestational effects, the progestins used in these menopausal hormone therapy formulations can have antiandrogenic, proandrogenic, glucocorticoid concentration, and antimineralocorticoid effects^11,12^ and are unlikely to provide direct evidence as to the potential association between endogenous progesterone and breast cancer risk. Mechanistic studies using cell culture, tissue culture, and preclinical models have associated progesterone with the development of breast cancer.^13^ To our knowledge, epidemiologic data are limited to 1 study reporting a null association between circulating progesterone levels and breast cancer risk among postmenopausal women.^14^ This study was limited by assay technology, with almost 30% of samples having undetectable levels of progesterone. Thus, the association of the presence of endogenous progesterone with breast cancer risk among postmenopausal women has been infrequently studied primarily owing to low levels of circulating progesterone, inadequate sensitivity of available assays,^15^ and/or large sample volumes required for clinical assays.

**Figure 1.**
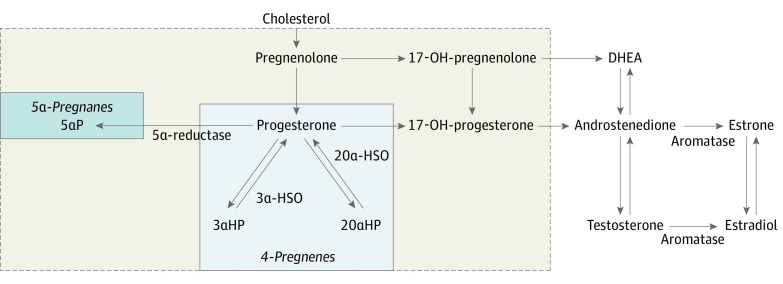
Schematic of the Synthesis of Sex Steroid Hormones From Cholesterol In normal breast tissue, pregnenes (progesterone is a pregnene) are the predominant compounds. All of the 4-pregnenes (not shown) can be irreversibly converted to 5α-pregnane via 5α-reductase. The 2 metabolites, 5α-dihydroprogesterone (5αP) and 3α-dihydroprogesterone (3αHP), demonstrate the greatest differences between breast tumor and nontumorous tissue in experimental models; the ratio of 5αP:3αHP is more than 10-fold higher in breast tumor tissues and 3-fold higher in circulation in mice that developed breast tumors vs mice without breast tumors. Actions of the progesterone metabolites are similar by age and by estrogen receptor status. The assay used measured the 7 progesterone-related compounds enclosed in the shaded area of the dashed-line box as follows: pregnenolone, progesterone, 17-OH-pregnenolone, 17-OH-progesterone, and select progesterone metabolites (5αP, 3αHP, and 20α-dihydroprogesterone). The estradiol concentration was measured previously, using an independent assay. 3α-HSO indicates 3α-hydroxysteroid oxidoreductase; DHEA, dehydroepiandrosterone; and 20-αHSO, 20α-hydroxysteroid oxidoreductase.

It has been hypothesized^16^ and laboratory studies have confirmed^17,18,19,20^ that breast cancer promotion may be associated with relative concentrations of progesterone metabolites, in particular the cancer-inhibiting 4-pregnenes (eg, 3α-dihydroprogesterone [3αHP]) and cancer-promoting 5α-pregnanes (eg, 5α-dihydroprogesterone [5αP]) (Figure 1). While the adrenal glands are the primary source of progesterone in postmenopausal women, smaller amounts are produced in adipose tissue, and local production of 3αHP and 5αP metabolites may occur in the breast.^17^ To our knowledge, no epidemiologic studies have evaluated the role of circulating progesterone metabolites and breast cancer risk. To address this data gap, we evaluated prediagnostic serum pregnenolone, progesterone, and their major metabolites in association with postmenopausal breast cancer risk in a large, established case-cohort study within the Breast and Bone Follow-up to the Fracture Intervention Trial (B ~ FIT).^21^ Existing data on circulating estrogens^21^ enabled evaluation of the relative concentration of progesterone to estradiol with breast cancer risk, given that, along with estrogen, progesterone is generally regarded as a proliferative agent in the healthy breast.^22^

## Methods

**Figure 2.**
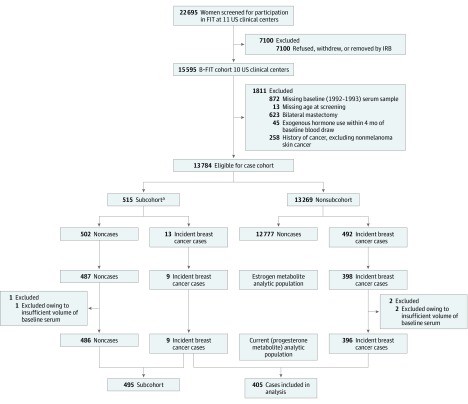
Case-Cohort Study Design The Breast and Bone Follow-up to the Fracture Intervention Trial (B ~ FIT) study was designed to follow-up women screened for the Fracture Intervention Trial (FIT) at 10 of the 11 participating FIT clinical centers. Vital status and cause of death of women who were screened (n = 15 595 women in B ~ FIT) was determined via linkage to the National Death Index Plus.^21^ From 2001 to 2004, surviving women who had been screened were contacted by mail and/or telephone and invited to complete a follow-up questionnaire that ascertained cancer diagnoses, other health outcomes, family history of cancer, detailed hormone use, preventive screening procedures, and reproductive surgeries that occurred since they completed the FIT health history questionnaire. The B ~ FIT questionnaire was completed by 64% of eligible women. Women included in the current case-cohort study were selected as depicted. Modified with permission from Dallal et al.^21^ IRB indicates institutional review board. ^a^Subcohort randomly selected within 10-year age and clinical center strata.

Details of the prospective case-cohort study on which this study is based have been described.^21^ In brief, the B ~ FIT cohort (n = 15 595) is a longitudinal cohort of postmenopausal women screened for participation in the Fracture Intervention Trial (FIT)^23^ from 1992 to 1993 at 10 of the 11 FIT clinical centers (Figure 2). Women in the B ~ FIT cohort included the following: (1) women screened for the FIT trial (screened only) who did not participate because they did not meet the eligibility for participation in FIT,^23^ (2) women randomized to participate in FIT, and (3) women invited to participate in an extension of the FIT Long-term Extension Trial (FLEX).^24,25,26^ The B ~ FIT questionnaire was completed by 64% of eligible women. All participants provided written informed consent; no financial compensation is provided. The B ~ FIT trial was approved by the institutional review board at each participating site and the National Cancer Institute; the present study was included in this approval. This study followed the Strengthening the Reporting of Observational Studies in Epidemiology (STROBE) reporting guideline for cohort studies.

Cancer incidence was based on questionnaire responses, with subsequent requests for medical record verification and/or cancer registry linkage at clinical sites in states with cancer registries or in Surveillance Epidemiology and End Results registry areas. Seventy-three percent of B ~ FIT participants resided in registry linkage areas, of which 29% were Surveillance, Epidemiology, and End Results registry areas.

Of the participants in the B ~ FIT cohort (n = 15 595), 1811 were excluded from possible selection into the case-cohort study (Figure 2). Of the remaining 13 784 eligible participants, 515 were randomly selected for the subcohort within 10-year age and clinical center strata. Additional exclusions were made owing to issues with sample vials, missing dates, and inability to confirm cancer diagnosis, resulting in 407 incident breast cancer cases and 496 women in the subcohort (9 incident breast cancers and 487 noncases) who were identified for a previous study measuring circulating estrogen and estrogen metabolite levels and breast cancer risk.^21^ More than 99% of these women had sufficient baseline serum remaining for the measurement of progesterone-related compounds (405 women with incident breast cancer and a subcohort of 495 postmenopausal women), which composed the present study population. Of the breast cancers included in the present analysis, 267 were diagnosed as invasive breast cancer and 54 as in situ cancer, with 84 missing information on cancer behavior. Among all breast cancer cases included in the present study, the mean (SD) time from sample collection to diagnosis was 5.6 (3.0) years (range, 0.2-12.6 years).

### Laboratory Assays

Stable isotope dilution high-performance liquid chromatography–tandem mass spectrometry (HPLC-MS/MS) was used to quantify pregnenolone, progesterone, and their major metabolite levels in serum: 17-hydroxypregnenolone, 17-hydroxyprogesterone, 3αHP, 5αP, and 20α-dihydroprogesterone. Details of the assay method have been published.^15^ Total estradiol levels were previously quantified using an independent HPLC-MS/MS assay.^21^

For all analytes in the assay, the lower limit of quantitation was 0.5 ng/dL and the lower limit of detection was 0.1 ng/dL. No samples in the present study had undetectable levels for any of the hormone concentrations measured. Laboratory coefficients of variation of deidentified duplicate samples within and across batches were less than 3.5% for all hormone concentrations measured. Intraclass correlation coefficients were greater than 0.97 (eAppendix in the Supplement). Progesterone assays were completed in July 2017; subsequent data analyses were conducted between July 15, 2017, and December 20, 2018.

### Statistical Analysis

Pregnenolone, progesterone, and their major metabolites were analyzed individually. The relative concentration of 5αP:3αHP was evaluated given experimental data supporting that relative changes in concentrations of 5αP reflect cancer-promoting properties and relative changes in concentrations of 3αHP reflect cancer-inhibiting properties.^17,20^ The relative concentration of progesterone and estradiol was also evaluated given in vivo data suggesting that both progesterone and estradiol are necessary to induce proliferation in the healthy breast.^22^ We evaluated the distribution of the hormones measured by the interval between blood draw and diagnosis, given that experimental studies suggested that circulating concentrations of 5αP are 3 times higher than 3αHP concentrations among women with breast cancer.^17,20^ Spearman rank correlations of the hormones measured were evaluated among women in the subcohort.

We evaluated possible deviations from linearity of the hormone-breast cancer association using a 5-knot spline with knots at the 10th, 25th, 50th, 75th, and 90th percentiles for each hormone based on the distribution in the subcohort. Hormones were modeled continuously, given that 5-knot splines did not suggest major deviations from linearity. Individual hormone measurements were scaled by their SD and ratio measures were analyzed without transformation. Cox proportional hazards regression with robust variance adjustment^27^ to account for the case-cohort design was used to estimate hazard ratios (HRs) and 95% CIs for the association between each hormone measurement or ratio and breast cancer risk. Age was used as the time measurement for all analyses. For the subcohort, women entered the analysis at age at baseline and contributed person-time until age at first diagnosis of breast cancer (event) or censoring (age at death or end of follow-up). Breast cancer cases not included in the subcohort entered the analysis 6 months before their age at diagnosis, contributing information only to their risk set. Exposure curves from survivor function plots were parallel, suggesting no deviation from proportional hazards.

Models were adjusted for a priori selected confounding factors, based on knowledge of the literature and study design considerations as follows: clinic (10 sites), trial participation status (only women who undergo screening, FIT, or FIT+FLEX), body mass index (continuous), and duration of prior menopausal hormone therapy use (never or <1, 1-4, 5-9, and ≥10 years).

To further test the hypothesis^20^ that relative concentrations of 5αP and 3αHP are associated with breast cancer risk, we explored associations modeling the cross-classification of 5αP and 3αHP in tertiles based on the distribution in the subcohort.

We evaluated potential effect modification of the progesterone and 5αP:3αHP associations with breast cancer risk by age (≤65 and >65 years at baseline), body mass index (<25.0, 25.0-29.9, ≥30.0 [calculated as weight in kilograms divided by height in meters squared]), prior oral contraceptive use (never, ever, and missing), and circulating estradiol level quintile. We maintained estradiol level quintiles given a prior B ~ FIT publication reporting increased breast cancer risk with the fifth compared with the first quintile of estradiol.^21^ Hazard ratios for breast cancer risk associated with increasing hormone concentration across categories of potential effect modifiers were estimated from stratified models, with significance assessed by a likelihood ratio test comparing a model with a multiplicative interaction term with a model without.

We conducted the following sensitivity analyses: censoring breast cancer cases that were not confirmed by medical record and/or cancer registry linkage (n = 48) and restricting the analytic study population to women who underwent screening (n = 694). Evaluation of hormone ratios, effect modification, and the stated sensitivity analyses were predefined in the analytic plan.

All *P* values were based on 2-sided tests and a nominal *P* value <.05 was considered statistically significant. Analyses were conducted in SAS, version 9.4 (SAS Institute Inc).

## Results

The current case-cohort study included 405 incident breast cancers and a subcohort of 495 postmenopausal women (Table 1, Figure 2). Participants’ mean (SD) age at blood draw was 67.2 (6.2) years and most were non-Hispanic white (384 [94.8%]). Mean (SD) age for women with breast cancer was 73 (6.4) years at diagnosis (range, 56-89). Among the subcohort, the correlations of hormone levels were primarily low to weak, with only a few analytes demonstrating strong correlation (correlation coefficient, 0.72 for progesterone with 17α-hydroxyprogesterone and 0.73 for progesterone with 20α-dihydroprogesterone) (eTable 1 in the Supplement).

**Table 1.** Demographic Characteristics of Women With Breast Cancer and Women in the Subcohort From a Case-Cohort Study Within B ~ FIT

Characteristic	No. (%)	
	Breast cancer cases (n = 405)	Subcohort (n = 495)
Age, y		
<65	148 (36.5)	187 (37.8)
≥65	257 (63.5)	308 (62.2)
Race/ethnicity		
Non-Hispanic white	384 (94.8)	469 (94.7)
Other	21 (5.2)	26 (5.3)
Group		
Screened	323 (79.8)	371 (74.9)
FIT	69 (17.0)	98 (19.8)
FIT + FLEX	13 (3.2)	26 (5.3)
Family history of breast cancer in first-degree relative		
No	316 (78.0)	419 (84.6)
Yes	81 (20.0)	61 (12.3)
Missing	8 (2.0)	15 (3.0)
BMI		
<25.0	147 (36.3)	210 (42.4)
25.0-29.9	138 (34.1)	156 (31.5)
>30.0	116 (28.6)	125 (25.3)
Missing	4 (1.0)	4 (0.8)
Alcohol use in the last month		
No	164 (40.5)	207 (41.8)
Yes	241 (59.5)	288 (58.2)
Parity		
Nulliparous	51 (12.6)	46 (9.3)
1	47 (11.6)	60 (12.1)
≥2	306 (75.6)	389 (78.6)
Missing	1 (0.2)	0
Oral contraceptive use		
Never	237 (58.5)	240 (48.5)
Ever	60 (14.8)	68 (13.7)
Missing	108 (26.7)	187 (37.8)
Duration of prior estrogen and/or progestin menopausal hormone therapy use, y		
Never or <1	296 (73.1)	378 (76.4)
1-4	61 (15.1)	65 (13.1)
5-9	19 (4.7)	27 (5.5)
≥10	26 (6.4)	24 (4.9)
Missing	3 (0.7)	1 (0.2)
Quintile of circulating estradiol, pg/mL		
Q1 (<6.30)	48 (11.9)	99 (20.0)
Q2 (6.30-8.61)	80 (19.8)	99 (20.0)
Q3 (8.62-12.64)	86 (21.2)	99 (20.0)
Q4 (12.65-18.70)	86 (21.2)	99 (20.0)
Q5 (>18.70)	105 (25.9)	99 (20.0)

Abbreviations: B ~ FIT, Breast and Bone Follow-up to the Fracture Intervention Trial; BMI, body mass index (calculated as weight in kilograms divided by height in meters squared); FIT, Fracture Intervention Trial; FLEX, FIT Long-term Extension Trial.

SI conversion factor: To convert estradiol level to picomoles per liter, multiply by 3.671.

The mean (SD) progesterone concentration among women in the subcohort 4.6 ng/dL (1.7) (to convert to picomoles per liter, multiply by 31.446) (eTable 2 in the Supplement). We did not observe increasing or decreasing progesterone metabolite concentrations as the time between blood draw and diagnosis decreased (eFigure 1 in the Supplement).

Women with higher circulating progesterone levels had a 16% increased risk of postmenopausal breast cancer per SD increase in progesterone (HR, 1.16; 95% CI, 1.00-1.35; *P* = .048) (Table 2). This association was linear in a 5-knot spline and strengthened (HR, 1.24; 95% CI, 1.07-1.43; *P* = .004) in models evaluating invasive breast cancer as the outcome (Table 2; eFigure 2 in the Supplement). The increased risk of breast cancer with higher levels of circulating progesterone also remained in sensitivity analyses evaluating medical record– and/or registry-confirmed breast cancer cases (HR, 1.18; 95% CI, 1.02-1.36; *P* = .03) and limited to women who were screened (HR, 1.22; 95% CI, 1.04-1.42; *P* = .01) (eTable 3 in the Supplement).

**Table 2.** Risk of Postmenopausal Breast Cancer per 1-SD Increase in Hormone Concentration or per 1-U Increase in Ratio of Hormone Concentrations

Factor	Breast cancer cases			
	All (n = 405)		Invasive (n = 267)	
	HR (95% CI)^a^	*P* value	HR (95% CI)^a^	*P* value
Pregnenolone	0.95 (0.82-1.11)	.53	0.96 (0.81-1.14)	.64
17-OH-pregnenolone	1.04 (0.91-1.19)	.55	1.07 (0.92-1.25)	.38
Progesterone	1.16 (1.00-1.35)	.048	1.24 (1.07-1.43)	.004
17-OH-progesterone	1.14 (0.99-1.31)	.06	1.18 (1.02-1.37)	.03
5αP	1.06 (0.93-1.20)	.39	1.04 (0.90-1.20)	.60
3αHP	1.13 (0.98-1.30)	.09	1.16 (0.99-1.36)	.06
20αHP	1.07 (0.94-1.21)	.33	1.10 (0.96-1.26)	.17
5αP:3αHP ratio	1.00 (0.97-1.04)	.85	1.00 (0.97-1.04)	.94
5αP:20αHP ratio	0.99 (0.87-1.12)	.84	0.97 (0.84-1.12)	.67
Progesterone:estradiol ratio	0.98 (0.94-1.02)	.33	0.99 (0.95-1.04)	.75

Abbreviations: 20αHP, 20α-dihydroprogesterone; 3αHP, 3α-dihydroprogesterone; 5αP, 5α-dihydroprogesterone; BMI, body mass index; HR, hazard ratio.

^a^ Hazard ratio per 1-SD increase in individual hormone concentration or per unit increase in ratio from proportional hazard regression model with robust variance estimates and adjusted for clinic site, trial group, BMI, and duration of prior estrogen and/or progestin menopausal hormone therapy use.

Higher levels of 5αP relative to 3αHP (5αP/3αHP) were not associated with risk of postmenopausal breast cancer (per unit increase in ratio: HR, 1.00; 95% CI, 0.97-1.04; *P* = .85) (Table 2). Associations evaluating concentrations of 5αP relative to 20α-dihydroprogesterone and progesterone relative to estradiol were also null. Among women in the lowest tertile (T) of 3αHP, the highest tertile of 5αP was associated with an almost doubling in breast cancer risk (T3 vs T1: HR, 1.96; 95% CI, 1.01-3.81; *P* = .04; *P* = .08 for interaction) compared with the lowest tertile (eTable 4 in the Supplement).

Progesterone associations with breast cancer and 5αP/3αHP-associations with breast cancer were not modified by age, body mass index, or prior oral contraceptive use (Table 3). The progesterone-breast cancer association was modified by quintile of estradiol as follows: higher vs lower circulating progesterone levels were associated with substantially reduced breast cancer risk among women within the lowest quintile of circulating estradiol (<6.30 pg/mL) (to convert estradiol level to picomoles per liter, multiply by 3.671) (HR per SD increase, 0.38; 95% CI, 0.15-0.95; *P* = .04), while higher vs lower circulating progesterone levels were associated with elevated breast risk cancer among women with higher quintiles of estradiol HRs: Q2, 1.32 (95% CI, 0.76-2.29); Q3, 1.27 (95% CI, 1.07-1.50); Q4, 1.19 (95% CI, 1.03-1.37); Q5, 1.23 (95% CI, 0.90-1.67) (*P* = .05 for interaction). Because we observed similar HRs across Q2 through Q5 (80% of women in our study), we combined these groups and found that higher circulating progesterone levels, specifically a 1-SD increase in progesterone level, was associated with an 18% increased risk of breast cancer (HR, 1.18; 95% CI, 1.04-1.35; *P* = .01; *P* = .04 for interaction) among women with circulating estradiol levels of 6.30 pg/mL or greater. The 5αP/3αHP-breast cancer association was not significantly modified by age, body mass index, oral contraceptive use, or circulating estradiol quintile.

**Table 3.** Risk of Breast Cancer per 1-SD Increase in Progesterone Concentration or per 1-U Increase in Ratio of Progesterone Metabolites

Factor	Progesterone			5αP/3αHP		
	HR (95% CI)^a^	*P* value	*P* Value for Interaction^b^	HR (95% CI)^a^	*P* value	*P* value for Interaction^b^
Age at baseline						
≤65	0.97 (0.66-1.45)	.90	.62	0.99 (0.93-1.05)	.70	.43
>65	1.21 (1.04-1.41)	.01		1.02 (0.98-1.07)	.37	
BMI						
<25.0	1.22 (1.05-1.41)	.01	.85	1.00 (0.94-1.05)	.85	.08
25.0-29.9	1.14 (0.89-1.46)	.30		0.95 (0.89-1.01)	.09	
≥30.0	1.28 (0.71-2.30)	.41		1.05 (0.99-1.11)	.08	
Prior oral contraceptive use						
Never	1.33 (1.14-1.55)	<.001	.15	0.98 (0.94-1.03)	.49	.76
Ever	0.92 (0.61-1.39)	.69		1.01 (0.93-1.09)	.84	
Missing	1.08 (0.67-1.74)	.76		1.00 (0.94-1.07)	.97	
Quintile of circulating estradiol, pg/mL						
Q1 (<6.30)	0.38 (0.15-0.95)	.04	.05	1.01 (0.85-1.20)	.92	.49
Q2 (6.30-8.61)	1.32 (0.76-2.29)	.32		0.92 (0.82-1.03)	.73	
Q3 (8.62-12.64)	1.27 (1.07-1.50)	.005		0.98 (0.92-1.05)	.54	
Q4 (12.65-18.70)	1.19 (1.03-1.37)	.67		1.03 (0.98-1.09)	.19	
Q5 (>18.70)	1.23 (0.90-1.67)	.19		1.02 (0.95-1.11)	.58	
Q2-Q5 combined	1.18 (1.04-1.35)	.01	.04	1.00 (0.96-1.04)	.88	.93

Abbreviations: 3αHP, 3α-dihydroprogesterone; 5αP, 5α-dihydroprogesterone; BMI, body mass index; HR, hazard ratio.

SI conversion factor: To convert estradiol level to picomoles per liter, multiply by 3.671.

^a^ Hazard ratio from proportional hazard regression model with robust variance estimates and adjusted for clinic site, trial group, BMI, and duration of prior estrogen and/or progestin menopausal hormone therapy use.

^b^ *P* value for interaction from likelihood ratio test.

## Discussion

In this prospective study, women with increased serum progesterone concentrations were at an increased risk of postmenopausal breast cancer. Higher levels of 5αP were not associated with breast cancer risk, except among women with the lowest levels of 3αHP. To our knowledge, this is the first population-based study to evaluate the association of novel progesterone metabolites with postmenopausal breast cancer risk. We noted effect modification of the progesterone-breast cancer association by estradiol level, whereby increased serum progesterone levels were associated with reduced breast cancer risks among women with very low total circulating estradiol levels and increased risks among women with higher circulating total estradiol levels.

In what appears to be the only prior study of progesterone and postmenopausal breast cancer risk published to date, progesterone was not associated with breast cancer risk.^14^ The mean concentration of progesterone in postmenopausal women in that study (mean, 4 ng/dL; range, 1.5 ng/dL [one-half the limit of detection of the assay] to 10 ng/dL), was consistent with the mean circulating progesterone concentration in our study population (mean, 4.6 ng/dL). However, the lowest concentrations measured in the previous study were below the limit of detection (3 ng/dL) of the radioimmunoassay used and represented almost one-third of the study population. It is likely that the reduced sensitivity of the progesterone assay available at the time the previous study was conducted may have limited the study’s ability to find an association. In the present study, no measures were below the limit of detection of the HPLC-MS/MS assay.

Several progesterone metabolites have been characterized in healthy breast and cancerous breast cancer tissue and broadly fall into 2 groups: 4-pregnenes, which are metabolites that retain their double bond, and 5α-pregnanes, which are metabolites in which 5α-reductase has reduced the double bond^16^ (Figure 1). Different relative distributions for 4-pregnenes and 5α-pregnanes have been demonstrated in healthy and cancerous breast tissue.^16,18^ In healthy breast tissue, the conversion of progesterone to 4-pregnene metabolites exceeds or predominates the conversion of progesterone to 5α-pregnane metabolites, whereas the opposite is true in breast cancer tissue. Our findings provide what appears to be little support for varying effects of different progesterone metabolites or their ratios measured in prediagnostic serum on breast cancer risk, which is inconsistent with the experimental data suggesting different relative concentrations in the 4-pregnenes and 5α-pregnanes measured as a ratio (5αP/3αHP) in breast tissue and circulation comparing established breast cancers with controls. However, when exploring the cross-classification of tertiles of 5αP and 3αHP concentrations, we noted an almost doubling in breast cancer risk comparing the highest third with the lowest third of 5αP concentrations among women with the lowest third of 3αHP concentrations. It is plausible that, at higher prediagnostic concentrations of 3αHP, the association with increasing 5αP concentration is either masked or not present when measured in circulation. However, we cannot rule out that variability in enzymatic activity of the hormones and metabolites may be influencing the observed association.

Progesterone’s actions are tissue specific and occur in concert with other hormones^22^; along with estrogen, progesterone is generally regarded as a proliferative agent in the healthy breast. Given that the women in our study were limited to those reporting no use of hormones within 4 months of the blood draw, our data provide support for a differential role of progesterone at physiologic levels that is possibly predicated on the level of circulating estradiol. In vivo studies support that both estradiol and progesterone may be responsible for cellular proliferation in the mammary gland and that progesterone is not sufficient to stimulate cellular proliferation in the absence of estradiol.^28^ Experimental data suggest the reliance on paracrine signaling for much of progesterone’s actions in breast tissue, but these data also show that *PR* transcription in mammary epithelium is dependent on interactions of estrogen with *ER-α*,^13,28,29^ further supporting the interdependency of progesterone and estradiol in mammary biology. We believe our data support that, at very low circulating estradiol levels, higher progesterone levels were associated with reduced breast cancer risk, suggesting that relatively higher progesterone levels may have a weak antimitotic effect on proliferation in the breast in this context. Although progesterone level was associated with increased breast cancer risk at higher circulating estradiol levels, further supporting that higher progesterone concentrations may increase risk via an enhancement of the mitotic effect of increased estradiol concentrations on breast tissue. While the interaction we observed is biologically plausible, replication in additional study populations is warranted.

### Strengths and Limitations

Our study has several strengths. The most important strength is the use of a highly sensitive and reproducible HPLC-MS/MS assay that quantifies hormone levels at concentrations relevant in postmenopausal women. Additional strengths include the use of prediagnostic serum, the prospective case-cohort design, the ability to stratify by circulating estradiol concentrations, and the large sample size.

The study also has limitations. A single blood sample may provide an imprecise average of long-term levels; however, a separate reproducibility study demonstrated moderate to high stability over 2 years (according to one of us [A.M.G.], written communication, March 20, 2020) in average intraclass correlation coefficients of all hormones measured (0.51, progesterone intraclass correlation coefficients, 0.84; 95% CI, 0.65-0.93 among postmenopausal women). We measured 7 hormones and included a moderate number of comparisons, suggesting that replication in future studies will be critical. We did not formally correct for multiple comparisons; however, using a Bonferroni-corrected *P* value of .005 (.05/10), only the positive association between progesterone concentrations and increased invasive breast cancer risk would have remained statistically significant.

The generalizability of our study may be limited, as the population comprises primarily women who were white and volunteered to be screened for participation in a clinical trial of medication to reduce fracture risk. However, as previously reported,^21^ the bone mineral density of women in our study is comparable to that of women from a nationally representative sample of US women. In addition, while we had information on tumor invasiveness for most cases, we were largely missing hormone receptor status, which may be relevant. However, 5αP and 3αHP are reported to act with equal efficacy regardless of the receptor status of the breast cancer cell line evaluated.^20^ Therefore, additional research to assess circulating associations by tumor characteristics (eg, ER/PR/ERBB2 [previously *HER2*] status) appears to be warranted.

## Conclusions

Our prospective data suggest that increased serum progesterone concentrations, measured using a highly sensitive HPLC-MS/MS assay, in postmenopausal women, appeared to be associated with an increased risk of breast cancer. There was a suggestion that increased risk was most apparent among women with moderate to high circulating total estradiol levels; at very low levels of estradiol, progesterone was associated with reduced breast cancer risk. While individual metabolites or their ratios were not associated with breast cancer risk, we noted an apparently increased risk with higher levels of 5αP among women with low levels of 3αHP. The possible identification of increased breast cancer risk with circulating levels of progesterone in postmenopausal women supports the need for additional research regarding its role in the cause of breast cancer overall and by tumor subtype. Further research is also needed to evaluate the role of progesterone metabolites and the interaction between progesterone and estradiol with breast cancer risk.

## Notes

Supplement. **eTable 1.** Spearman Ranked Correlation of Hormone Measurements and Ratios Among Subcohort (N = 495) Measured in B~FIT **eTable 2.** Distribution of Progesterone and Progesterone-Related Hormones Among Breast Cancer Case and Subcohort Members in ng/dL: Case-Cohort Study Within B~FIT **eFigure 1.** Progesterone Concentration (ng/dL) by Time Between Blood Draw and Diagnosis **eFigure 2.** Five-Knot Spline of the Association Between Circulating Progesterone and Postmenopausal Breast Cancer Risk **eTable 3.** Risk of Postmenopausal Breast Cancer per Standard Deviation Increase in Hormone Concentration or per One-Unit Increase in Ratio of Hormone Concentrations; Case-Cohort Study Within B~FIT **eTable 4.** Breast Cancer Risk in Models Evaluating the Cross-Classification of 5α-Dihydroprogesterone (5αP) and 3α-Dihydroprogesterone (3αHP) **eAppendix.** Supplemental assay methods. Details of HPLC-MS/MS assay validation and quality control
