# Supplementary material for: Association of Circulating Progesterone With Breast Cancer Risk Among Postmenopausal Women
Source: JAMA Netw Open. 2020 Apr 24;3(4):e203645. doi: 10.1001/jamanetworkopen.2020.3645 (PMC7182797; doi:10.1001/jamanetworkopen.2020.3645)
Supplement: Supplement. — eTable 1. Spearman Ranked Correlation of Hormone Measurements and Ratios Among Subcohort (N = 495) Measured in B~FIT eTable 2. Distribution of Progesterone and Progesterone-Related Hormones Among Breast Cancer Case and Subcohort Members in ng/dL: Case-Cohort Study Within B~FIT eFigure 1. Progesterone Concentration (ng/dL) by Time Between Blood Draw and Diagnosis eFigure 2. Five-Knot Spline of the Association Between Circulating Progesterone and Postmenopausal Breast Cancer Risk eTable 3. Risk of Postmenopausal Breast Cancer per Standard Deviation Increase in Hormone Concentration or per One-Unit Increase in Ratio of Hormone Concentrations; Case-Cohort Study Within B~FIT eTable 4. Breast Cancer Risk in Models Evaluating the Cross-Classification of 5α-Dihydroprogesterone (5αP) and 3α-Dihydroprogesterone (3αHP) eAppendix. Supplemental assay methods. Details of HPLC-MS/MS assay validation and quality control [file jamanetwopen-3-e203645-s001.pdf]

Supplementary Online Content

Trabert B, Bauer DC, Buist DSM, et al. Association of circulating progesterone with breast cancer risk among postmenopausal women. *JAMA Netw Open*. 2020;3(4):e203645. doi:10.1001/jamanetworkopen.2020.3645

eTable 1. Spearman Ranked Correlation of Hormone Measurements and Ratios Among Subcohort (N=495) Measured in B~FIT

eTable 2. Distribution of Progesterone and Progesterone-Related Hormones Among Breast Cancer Case and Subcohort Members in ng/dL: Case-Cohort Study Within B~FIT

eFigure 1. Progesterone Concentration (ng/dL) by Time Between Blood Draw and Diagnosis

eFigure 2. Five-Knot Spline of the Association Between Circulating Progesterone and Postmenopausal Breast Cancer Risk

eTable 3. Risk of Postmenopausal Breast Cancer per Standard Deviation Increase in Hormone Concentration or per One-Unit Increase in Ratio of Hormone Concentrations; Case-Cohort Study Within B~FIT

eTable 4. Breast Cancer Risk in Models Evaluating the Cross-Classification of 5α-Dihydroprogesterone (5αP) and 3α-Dihydroprogesterone (3αHP)

eAppendix. Supplemental assay methods: Details of HPLC-MS/MS assay validation and quality control

This supplementary material has been provided by the authors to give readers additional information about their work.

© 2020 Trabert B et al. *JAMA Network Open.*

eTable 1. Spearman ranked correlation of hormone measurements and ratios among subcohort (n=495) measured in B~FIT.

	Pregnenolone	17-OH-Pregnenolone	Progesterone	17-OH-Progesterone	5αP	3αHP	20αHP	Total Estradiol	5αP/3αHP ratio	5αP/20αHP ratio	Progesterone/Estradiol ratio
Pregnenolone	1.00										
17-OH-Pregnenolone	0.16	1.00									
Progesterone	0.04	0.19	1.00								
17-OH-Progesterone	0.08	***0.34***	***0.72***	1.00							
5α-Dihydroprogesterone (5αP)	0.14	-0.01	-0.03	0.01	1.00						
3α-Dihydroprogesterone (3αHP)	0.14	0.09	-0.01	0.05	0.14	1.00					
20α-Dihydroprogesterone (20αHP)	0.01	0.11	***0.73***	***0.39***	-0.07	-0.03	1.00				
Total Estradiol	-0.02	0.00	-0.02	0.02	-0.06	0.00	0.15	1.00			
5αP/3αHP ratio	0.01	-0.08	-0.02	-0.04	***0.58***	***-0.70***	-0.03	-0.04	1.00		
5αP/20αHP ratio	0.10	-0.08	***-0.40***	***-0.20***	***0.85***	0.13	***-0.56***	-0.13	0.48	1.00	
Progesterone/Estradiol ratio	0.03	0.07	***0.37***	***0.24***	0.05	-0.01	0.12	***-0.92***	0.04	-0.03	1.00

*Correlations significant at p-value <0.0001 are bold and italicized.

© 2020 Trabert B et al. *JAMA Network Open.*

eTable 2. Distribution of progesterone and progesterone-related hormones among breast cancer case and subcohort members in ng/dL: case-cohort study within B~FIT.

	Cases (N=405)		Subcohort (N=495)	
Hormone (ng/dL)	**Mean**	**SD**	**Mean**	**SD**
Pregnenolone	15.9	7.7	16.2	7.2
17-OH-Pregnenolone	95.9	44.6	95.6	43.9
Progesterone	4.7	2.7	4.6	1.7
17-OH-Progesterone	16.0	14.5	15.2	10.8
5α-Dihydroprogesterone (5αP)	8.0	3.6	7.8	3.5
3α-Dihydroprogesterone (3αHP)	2.5	1.3	2.4	1.2
20α-Dihydroprogesterone (20αHP)	5.0	1.6	4.9	1.6
Hormone (pmol/L)				
Pregnenolone	503	244	512	229
17-OH-Pregnenolone	2884	1343	2876	1320
Progesterone	150	87	146	53
17-OH-Progesterone	485	439	459	328
5α-Dihydroprogesterone (5αP)	251	114	247	112
3α-Dihydroprogesterone (3αHP)	78	41	75	38
20α-Dihydroprogesterone (20αHP)	157	50	154	52
Total estradiol	55	40	50	53
Ratio of molar concentration				
5αP/3αHP ratio	4.6	4.4	4.4	3.8
5αP/20αHP ratio	1.7	0.9	1.8	1.0
Progesterone/estradiol ratio	4.0	3.7	4.6	4.2
Progesterone levels (ng/dL) by quintile of circulating estradiol				
Estradiol Q1 (<6.30 pg/mL)	4.2	0.9	4.5	1.5
Estradiol Q2 (6.30-8.61 pg/mL)	4.7	1.5	4.6	1.3
Estradiol Q3 (8.62-12.64 pg/mL)	5.3	5.4	4.5	1.3
Estradiol Q4 (12.65-18.70 pg/mL)	4.5	1.0	4.7	2.2
Estradiol Q5 (>18.70 pg/mL)	4.7	1.5	4.6	2.0

SD = standard deviation

© 2020 Trabert B et al. *JAMA Network Open.*

eFigure 1. Progesterone metabolite concentration (ng/dL) by time between blood draw and diagnosis.

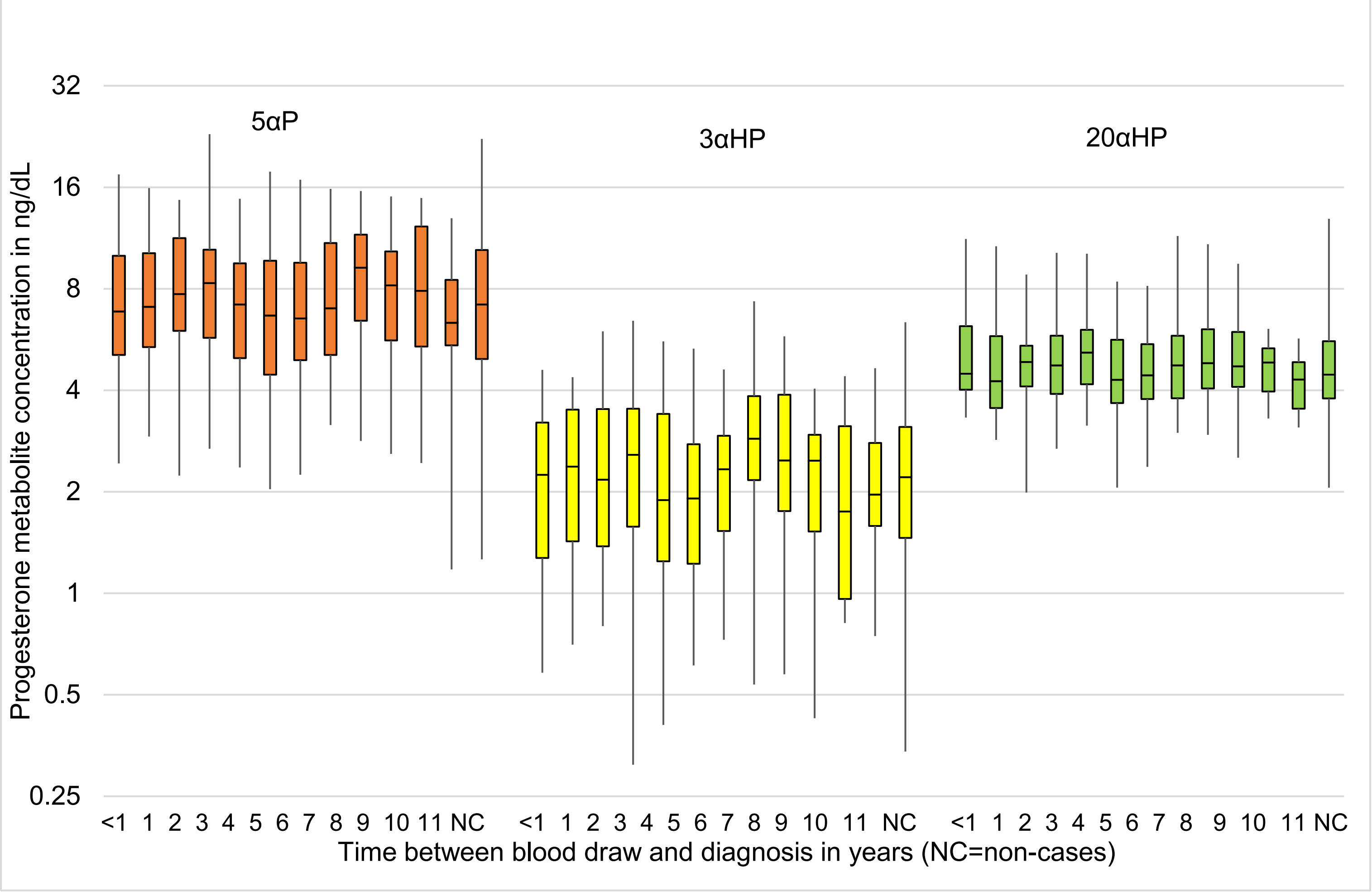

eFigure 1 legend: Results from the experimental study by Wiebe et al. support that circulating concentrations of 5αP are three times higher than 3αHP among women with breast cancer. Thus, it could be suggested then that the difference between the progesterone metabolites should increase in blood samples collected closer to diagnosis, although this has not been tested. In the current observational study, we did not see any deviation across time to diagnosis· We did observe that the 5αP concentrations are about three times the 3αHP concentration irrespective of time between blood draw and breast cancer diagnosis. NC = non-cases

© 2020 Trabert B et al. *JAMA Network Open.*

eFigure 2. Five-knot spline of the association between circulating progesterone and postmenopausal breast cancer risk.

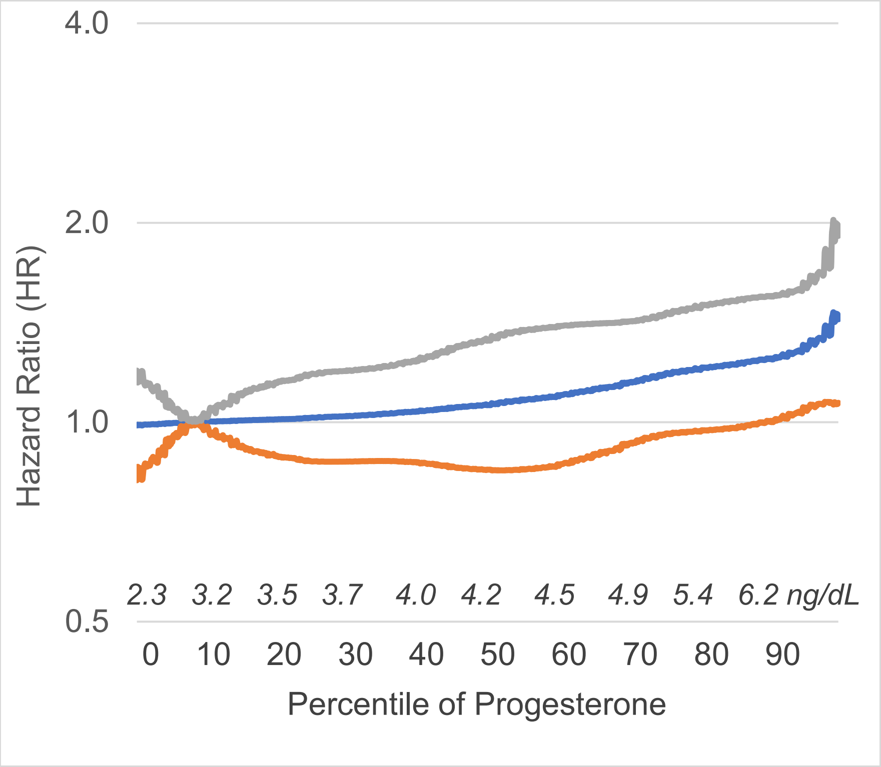

eFigure 2 Legend: Blue line=HR, Orange line=lower 95% CI, Grey line=upper 95% CI. X-axis reported as percentile of circulating progesterone (the corresponding progesterone concentration for the percentile cutpoint is reported in italics).

© 2020 Trabert B et al. *JAMA Network Open.*

eTable 3. Risk of postmenopausal breast cancer per standard deviation increase in hormone concentration or per one-unit increase in ratio of hormone concentrations; case-cohort study within B~FIT.

	Medical record and/or registry confirmed cases (n=357 cases)			Screenees only (N=694, n=323 cases)		
	**HR***	**(95% CI)**	**P Value**	**HR***	**(95% CI)**	**P Value**
Pregnenolone	0.94	(0.80-1.10)	0.43	0.86	(0.73-1.02)	0.08
17-OH-Pregnenolone	1.04	(0.90-1.19)	0.62	1.02	(0.88-1.20)	0.78
Progesterone	1.18	(1.02-1.36)	0.03	1.22	(1.04-1.42)	0.01
17-OH-Progesterone	1.16	(1.01-1.33)	0.04	1.25	(1.06-1.48)	0.01
5α-Dihydroprogesterone (5αP)	1.06	(0.93-1.21)	0.39	1.05	(0.91-1.21)	0.51
3α-Dihydroprogesterone (3αHP)	1.14	(0.99-1.32)	0.08	1.18	(1.00-1.40)	0.06
20α-Dihydroprogesterone (20αHP)	1.06	(0.93-1.20)	0.42	1.03	(0.90-1.19)	0.66
5αP/3αHP ratio	1.01	(0.97-1.04)	0.71	1.00	(0.96-1.04)	0.98
5αP/20αHP ratio	1.01	(0.89-1.14)	0.93	0.99	(0.86-1.13)	0.83
Progesterone/estradiol ratio	0.98	(0.94-1.02)	0.39	1.00	(0.95-1.04)	0.89

*Hazard Ratio (HR) per standard deviation increase in individual hormone concentration or per unit increase in ratio from proportional hazard regression model with robust variance estimates and adjusted for clinic site, trial group, BMI, and duration of prior estrogen and/or progestin menopausal hormone therapy use.

© 2020 Trabert B et al. *JAMA Network Open.*

eTable 4. Breast cancer risk in models evaluating the cross-classification of 5α-Dihydroprogesterone (5αP) and 3α-Dihydroprogesterone (3αHP).

	3αHP			
	**Tertile (T)1 (<1.72 ng/dL)**	**T2 (1.72-2.79 ng/dL)**	**T3 (>2.79 ng/dL)**	
**5αP**	**HR (95% CI)**	**HR (95% CI)**	**HR (95% CI)**	**P-Intx†**
T1 (<5.62 ng/dL)	ref	ref	ref	0.08
T2 (5.62-9.12 ng/dL)	1.68 (0.92-3.09)	0.72 (0.38-1.38)	1.11 (0.62-1.99)	
T3 (>9.12 ng/dL)	1.96 (1.01-3.81)	0.58 (0.32-1.06)	1.10 (0.62-1.95)	
P-Trend	0.04	0.08	0.76	

*Hazard Ratio (HR) from proportional hazard regression model with robust variance estimates and adjusted for clinic site, trial group, BMI, and duration of prior estrogen and/or progestin menopausal hormone therapy use.
†P interaction (P-Intx) from likelihood ratio test.

© 2020 Trabert B et al. *JAMA Network Open.*

eAppendix. Supplemental assay methods: Details of HPLC-MS/MS assay validation and quality control

The calibration curves for the progesterone and progesterone-related hormones (PM for short) measured in this study were linear over 1000-fold concentration range with linear regression correlation coefficients greater than 0.998. The lower limit of quantitation (LLOQ) in the present PM assay was defined as the lowest concentration of an analyte in a sample that could be determined with acceptable precision and accuracy defined as intra- and inter-batch coefficient of variation within 15% and measured analyte values within 85% to 115% of known target values under the conditions of the described assay method [1,2]. The LLOQ for all hormones measured in our PM LC-MS/MS assay are 0.5 ng/dL, and lower limit of detection (LOD) are 0.1 ng/dL. The PM HPLC-MS/MS assay accuracy was measured as the percent recovery of the known added amount of analyte in spiked samples [1,2]. High specificity of our assay was achieved through triple-quad selective reaction monitoring coupled with high resolution chromatography.

Laboratory coefficients of variation (CVs) of blinded duplicate quality control (QC) samples within and across batches were as follows: pregnenolone 2.6%; 17-OH-pregnenolone 1.7%; progesterone 3.1%; 17-OH-progesterone 3.5%; 5α-dihydroprogesterone 1.8%; 3α-dihydroprogesterone 3.3%; 20α-dihydroprogesterone 2.7%. Intraclass correlation coefficients (ICCs) were as follows: pregnenolone 0.99; 17-OH-pregnenolone 0.99; progesterone 0.99; 17-OH-progesterone 0.98; 5α-dihydroprogesterone 0.99; 3α-dihydroprogesterone 0.99; 20α-dihydroprogesterone 0.99. The mean value of each set of QC replicates distributed within and across batches and corresponding CV were as follows:

	**QCA**	**QCB**	**QCC**	**QCD**	**QCE**	**QCF**	**QCG**	**QCH**
Hormone (ng/dL)	**Mean (CV%)**	**Mean (CV%)**	**Mean (CV%)**	**Mean (CV%)**	**Mean (CV%)**	**Mean (CV%)**	**Mean (CV%)**	**Mean (CV%)**
Pregnenolone	5.1 (1.0)	6.8 (1.7)	45.4 (3.9)	19.9 (3.3)	15.3 (0.6)	7.8 (2.3)	19.5 (2.5)	22.3 (3.6)
17-OH-Pregnenolone	175.0 (0.6)	94.7 (1.6)	813.2 (1.1)	164.8 (2.5)	80.1 (0.3)	75.4 (1.9)	173.9 (0.7)	181.0 (1.8)
Progesterone	1.7 (2.2)	3.0 (2.1)	4.2 (2.5)	3.5 (4.9)	4.0 (0.8)	3.4 (5.1)	4.2 (3.0)	561.0 (1.7)
17-OH-Progesterone	10.0 (2.1)	10.6 (3.1)	22.1 (3.5)	10.2 (4.5)	14.3 (0.7)	27.3 (4.8)	9.9 (1.8)	29.0 (3.8)
5α-Dihydroprogesterone (5αP)	4.7 (0.7)	4.2 (0.8)	15.1 (2.8)	5.8 (2.1)	5.2 (0.5)	4.8 (2.3)	9.9 (1.4)	52.1 (2.4)
3α-Dihydroprogesterone (3αHP)	1.2 (3.8)	0.4 (1.0)	2.2 (3.4)	0.5 (2.5)	3.2 (1.1)	1.2 (6.7)	0.6 (1.7)	2.1 (3.5)
20α-Dihydroprogesterone (20αHP)	2.5 (2.0)	2.4 (3.2)	10.0 (2.4)	4.3 (3.6)	4.7 (0.8)	3.0 (1.9)	6.4 (1.8)	153.8 (2.3)

1. Swartz ME, Krull IS. Analytic method validation and development, a primer. Marcel Dekker, Inc. New York, 1997
2. Guidance for Industry: Bioanalytical Method Validation, US HHS FDA May 2018, https://www.fda.gov/media/70858/download

© 2020 Trabert B et al. *JAMA Network Open.*
